# Cognitive Frequency-Hopping Waveform Design for Dual-Function MIMO Radar-Communications System

**DOI:** 10.3390/s20020415

**Published:** 2020-01-11

**Authors:** Yu Yao, Xuan Li, Lenan Wu

**Affiliations:** 1 School of Information Engineering, East China Jiaotong University, Nanchang 330031, China; lixuan0799@outlook.com; 2 School of Information Science and Engineering, Southeast University, Nanjing 210096, China; wuln@seu.edu.cn

**Keywords:** multiple-input multiple-output (MIMO), frequency-hopping code, dual-function radar-communications, information embedding, mutual information (mi), waveform optimization

## Abstract

A frequency-hopping (FH)-based dual-function multiple-input multiple-output (MIMO) radar communications system enables implementation of a primary radar operation and a secondary communication function simultaneously. The set of transmit waveforms employed to perform the MIMO radar task is generated using FH codes. For each transmit antenna, the communication operation can be realized by embedding one phase symbol during each FH interval. However, as the radar channel is time-variant, it is necessary for a successive waveform optimization scheme to continually obtain target feature information. This research work aims at enhancing the target detection and feature estimation performance by maximizing the mutual information (MI) between the target response and the target returns, and then minimizing the MI between successive target-scattering signals. The two-step cognitive waveform design strategy is based upon continuous learning from the radar scene. The dynamic information about the target feature is utilized to design FH codes. Simulation results show an improvement in target response extraction, target detection probability and delay-Doppler resolution as the number of iterations increases, while still maintaining high data rate with low bit error rates between the proposed system nodes.

## 1. Introduction

The multiple-input multiple-output (MIMO) radar [1,2,3] is a significant technique owing to the enhancement in performance it provides over the traditional radar systems with a single transmit antenna. The transmit waveforms are detected by a matched filter bank in the MIMO radar receiver. Making use of the knowledge of the propagation channels, a superior spatial resolution can be achieved. Furthermore, a MIMO radar has several advantages such as prominent interference rejection capability, enhanced parameter estimation performance, and better flexibility for transmit waveform optimization [4,5,6]. Several works in the literature discussed the subject of MIMO waveform optimization [7,8,9,10,11]. In [7], the authors considered the joint design of both transmit waveforms and receive filters for a collocated MIMO radar with the existence of signal-dependent interference and white noise. The design problem is formulated into a maximization of the signal-to-interference-plus-noise ratio (SINR), including various constraints on the transmit waveforms. Stoica and Li designed the covariance matrix of the transmit waveforms to control the spatial power [8]. However, the cross correlation between the transmit waveforms at a specific target range is minimized. Several research works [12,13,14,15] designed the transmit waveform directly instead of just the covariance matrix. The work of [13] considered prior knowledge of the target impulse response (TIR) and utilized the information to select the transmit waveforms which optimize the mutual information (MI) between the target echoes and the TIR. The work of [15] investigated the problem of the spectrally compatible waveform design for MIMO radar in the presence of multiple targets and signal-dependent interference. A new method was proposed to deal with a more general problem, i.e., designing a spectrally compatible waveform for multiple targets, by minimizing the waveform energy of the overlayed space-frequency bands. The waveform optimization which employs prior knowledge of TIR is also implemented in the single-input multiple-output (SIMO) radar system [16].

The work of [17] derived MIMO radar ambiguity functions. San Antonio and Fuhrmann discussed some properties of the MIMO radar ambiguity function, which provide several ideas for MIMO waveform optimization [17]. The frequency-hopping (FH) sequences presented in [18] were used in MIMO radar configuration [19,20]. The FH orthogonal transmit waveforms discussed in [18] are initially considered for multiuser radar system. Furthermore, [18] employed the FH codes to minimize the peaks in the cross correlation functions of the transmit waveforms as much as possible. However, in the multiuser radar scenario, each operator activates its individual system. This is different from the MIMO radar system where the receiver antennas can cooperate to resolve the target responses. The FH sequences, which were designed by Chen and Yang, optimized the MIMO ambiguity function.

Based on their characteristics of being easily produced and modulus constant, FH codes are considered a good choice for the MIMO radar waveforms. A new scheme for optimizing the MIMO radar waveforms was provided in [19]. The scheme makes the energy of the MIMO ambiguity function spread in the range and angular dimensions evenly, as well as decreases the sidelobes in the MIMO radar ambiguity function. The work of [19] also designed optimal FH waveforms, which had separate FH codes and amplitudes, for a collocated MIMO radar system. The joint design problem can be solved by using game theory, provided by [20]. The authors considered the two objective functions, corresponding to FH codes and amplitudes separately, as two interacting players. By this concept, the joint optimization scheme obtained better integrated code and amplitude matrices that can improve performance much better than separate designs. 

The works of [21,22,23,24,25,26] indicated a possibility of employing the radar-communications integration concept to solve the lack of radio frequency (RF) spectrum. Efficient utilization of shared bandwidth between wireless communications and radar can be achieved by using dynamic frequency allocation. For example, using dynamic frequency allocation is a way to make shared bandwidth between wireless communications and radar possess more efficient utilization. The work of [23] proposed a novel dual-function radar-communications (DFRC) strategy to embed quadrature amplitude modulation (QAM) based communication information in the radar waveforms by exploiting sidelobe control and waveform diversity. In [27,28], the authors proposed a novel distributed DFRC MIMO system capable of simultaneously performing radar and communication tasks. The distributed DFRC MIMO system performs both objectives by optimizing the power allocation of the different transmitters in the DFRC system. The proposed strategy can serve multiple communication receivers located in the vicinity of the distributed DFRC MIMO system. Numerous recent studies [29,30,31] considered that the developing concept of DFRC is secondary to the main radar task. Communication source embedding into the illumination of MIMO radar system is realized using waveform diversity, sidelobe control, or the time-modulated array technique, which was studied in [30]. Hassanien and Himed presented a signaling strategy for communication source embedding into the illumination of FH-based MIMO radar system [32]. The main principle behind the signaling strategy is to embed phase modulation (PM)-based symbols by using phase rotating the FH pulses. The phase shift is implemented to each transmit FH waveform of the MIMO radar system. The PM-based symbol embedding does not influence the function of the MIMO radar system, which uses the FH waveforms.

The investigation on cognitive radar waveform optimization has received a lot of interest [33,34,35]. To further enhance the performance of the TIR estimation in a time-varying target scene, the transmitted signal parameters should be constantly adjusted. Then, updated knowledge about the time-varying target scene is employed to allocate fundamental resources like transmitted signal parameters in a cognitive mode [34]. A new strategy for optimizing the waveforms of a cognitive radar was presented in [34]. The aim is to enhance the performance of target estimation by minimizing the mean-square error (MSE) of the estimates of target scattering coefficients (TSC) based on Kalman filtering and then minimizing MI between the radar target echoes at successive time instants. However, there is also an increase in the computational load due to the Kalman filtering step in the waveform optimization. Such a cognitive radar system cannot be used in real applications to address the environmental sensing issues. To improve the performance of the target parameters estimation and classification, the pioneering study by Bell in [36] developed an information-theoretic method for the radar waveforms optimization. The authors in [37,38,39] extended the information-theoretic method by maximizing the MI between the target response and the target-scattering signals as a waveform design criterion in the MIMO radar system. The work of [40] proposed an innovative method to designing the transmit signal of cognitive MIMO radar system, which combines the signal optimization and selection processes. The works of [41,42,43,44] present a signaling scheme for information embedding into the illumination of the radar using FH pulses. An FH-based joint radar-communication system enables implementing a primary radar operation and a secondary communication function simultaneously. Then, the authors consider the problem of radar codes optimization under a peak-to-average-power ratio (PAR) and an energy constraint. However, a time-variant radar scenario is not considered.

Based on the points discussed above, it is interesting to discuss the performance of an adaptive dual-function MIMO radar-communications system that combines the adaptive FH waveform optimization scheme stated in [38] and PM-based information embedding strategy stated in [31]. To further adapt to the dynamic radar environment, we consider the problem of adaptive waveforms design and propose a two-step waveform optimization scheme, which provides better target detection performance and high data-rate communication capability between the proposed dual-function systems. The proposed scheme is summarized as follows:

Step 1. Waveform Design: this part includes the design of the FH waveform for the individual MIMO transmit antennas. The primary goal is to maximize the MI between the target-scattering signal and the estimated target response. The method ensures that the target-scattering signal at each time instant is dependent on the target response. 

Step 2. Waveform Selection: after the best waveform ensemble is gained, part two is to choice the suitable PM-based FH sequences for emission. The principle of this module is to minimize the MI between consecutive target scattering signals. This part ensures that we constantly obtain target returns that become independent of each other in time, with the purpose of achieving more information about the target characteristics at each time instant of reception.

The premise of the FH waveform optimization scheme is channel estimation. The target feature estimation can be performed by the MIMO radar receiver through observations implemented in the previous time instant. A feedback loop enables the delivery of the estimates to the dual-function MMO radar-communications transmitter. At a result, the optimization strategy allows the MIMO radar transmitter to constantly adjust FH codes to suit the time-varying channel scene.

We choose the FH waveform for the following reasons:

(1) The channel environment is complicated to wireless communications due to densely populated scatterers. However, FH waveforms are immune to multipath channel fading under the circumstances.
(2) The FH waveforms are robust to antagonistic environments by offering low interception probability. Furthermore, FH waveforms are immune to clutter interference.
(3) The constant-modulus waveforms have the property of high transmission power efficiency. FH waveforms enjoy the constant-modulus feature and are easy to generate.

The primary innovations of our work are summarized as follows:

(1) We define the PM-based FH waveforms in dual-function MIMO radar-communications configuration and derive the associated MIMO ambiguity function;
(2) We develop a two-step waveform optimization scheme in the adaptive PM-based dual-function MIMO radar-communications framework;
(3) We evaluate the performance of the proposed scheme in terms of target response estimation, delay-Doppler resolution and communication symbol error rate (SER).
(4) We compare the proposed scheme with other radar systems through analysis of the target detection and receiver operating characteristics (ROC) in an interference noise environment.

The organization of this paper is as follows. In Section 2, we describe the dual-function MIMO radar-communications system and the PM-based FH signal model. In Section 3, we derive the MIMO radar ambiguity function of the PM-based FH waveform. In Section 4, we present an adaptive approach to optimizing the proposed information embedding waveform. The transmit waveforms are designed at step 1 of the algorithm and selected based on the criterion presented in step 2. The simulation results demonstrating the proposed scheme are presented in Section 5. Finally, our conclusions and directions for possible future work are drawn in Section 6.

Throughout this paper, the following notations will be used. We use boldface lowercase letters and boldface uppercase letters to denote vectors and matrices, respectively; ${\left(.\right)}^{*}$ to denote the complex conjugate operation; ${\left(.\right)}^{T}$ to denote the transpose operation; ${\left(.\right)}^{H}$ to denotes the Hermitian operation; ⊗ to denote the Kronecker product; $I_{MN}$ to denote the identity matrix of size MN×MN; Angle(⋅) to denote the angle of a complex number.

## 2. System Configuration and Signal Model

### 2.1. Phase Modulation (PM)-Based Frequency Hopping (FH) Waveforms

A PM-based method for embedding information into the radar emission was recently proposed in [31]. To deliver a finite number of binary bits per radar pulse, the PM-based method maps the binary data into a phase symbol that belongs to a phase dictionary of an appropriate size. During each radar pulse, the PM-based method embeds one phase symbol into the radar emission toward the intended communication direction. At the communication receiver, a phase detector is used to detect the embedded symbol and, subsequently, deciphers the corresponding binary sequence. Unlike the amplitude modulation (AM) and amplitude shift keying (ASK) methods [30], the PM-based method offers the ability to embed information toward communication receivers, regardless of whether they are located within the sidelobe or the main lobe.

Since target detection is the main task of the dual-function radar-communications system, the transmit waveform should be considered primarily based on the requirements of the radar function. One fundamental requirement of the radar function is the high efficiency of transmitted power. So constant-modulus waveforms are selected. FH waveform enjoys the constant-modulus feature and is easy to generate. Furthermore, FH pulse waveform is immune to multipath channel fading and clutter interference under the circumstances.

The configuration for a joint radar-communications system and the PM-based FH signal model was developed in [44]. In this section, we follow the methods of [44] and further develop a PM-based dual-function MIMO radar-communications system. We express the MIMO FH waveforms as

(1) $$\phi_{m}\left(t\right)=\sum_{q=1}^{Q}e^{j2\pi c_{m,q}\Delta ft}u\left(t-\Delta t\right),m=1,\ldots ,M$$

where $c_{m,q},m=1,\ldots ,M;q=1,\ldots ,Q$ describes the FH code, M and Q denote the number of transmit antennas and the length of FH code, respectively. Δf and Δt respectively denote the frequency step and the hopping interval duration, and

(2) $$u\left(t\right)=\{\begin{array}{cc} 1, & 0<t<\Delta t\\ 0, & otherwise \end{array}$$

The duration of a pulse is expressed as $T_{0}=Q\Delta t$. We assume that the code is $c_{m,q}\in \left\{1,\ldots ,J\right\}$, where J denotes a predefined value. As a result, the bandwidth of the radar pulse can be approximately denoted by JΔf. To realize transmit waveform orthogonality, FH code should be designed to meet the following requirement $c_{m,q}=c_{m^{\prime },q},\forall q,m=m^{\prime }$. Several papers proposed FH waveform optimization for MIMO systems (see [19]; and references therein). Then, we propose that a dual-function MIMO radar-communications system with phase symbols yields the extended virtual data model at the radar receiver. We also present the information-embedding scheme and the associated transmission rate. Let $\left\{\Omega_{m,q}\in \left[0,2\pi \right]\right\},m=1,\ldots ,M,q=1,\ldots ,Q$ be a set of MQ PM-based symbols. Hence, the set of FH waveforms is expressed as:

(3) $$x_{m}\left(t\right)=\sum_{q=1}^{Q}e^{j\Omega_{m,q}}e^{j2\pi c_{m,q}\Delta ft}u\left(t-\Delta t\right),m=1,\ldots ,M$$

In (3), Δt should be designed to meet the requirement:

(4) $$\int_{0}^{\Delta t}e^{j2\pi c_{m,q}\Delta ft}e^{-j2\pi c_{m^{\prime },q^{\prime }}\Delta ft}dt=0,m=m^{\prime },q=q^{\prime }$$

**Figure 1:**
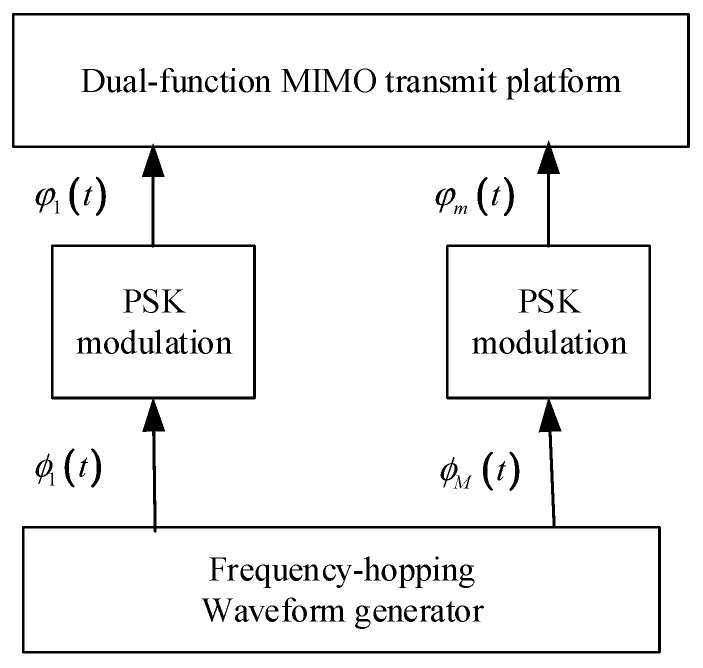
Illustrative diagram of a dual-function multiple-input multiple-output (MIMO) transmit platform.

By using (1) and the orthogonality between the FH waveforms stated in (4), it is easy to prove that PM-based FH waveforms $x_{m}\left(t\right),m=1,\ldots ,M$ are also orthogonal. The set of transmitted waveforms is presented in Figure 1.

The N×1 vector of the target scattering signals at the output of the radar receive antenna array can be expressed as:

(5) $$y\left(t,i\right)={Η}_{}^{T}\left(i\right)x\left(t,i\right)+n\left(t,i\right)$$

where *i* describes the index. $Η\left(i\right)={\left[h_{m,n}^{i}\right]}_{M\times N}$ indicates the target response matrix during the *i*-th radar scan. $h_{m,n}$ stands for the channel coefficient between the *m*-th transmit element and the *n*-th receive element. $x\left(t,i\right)={\left[x_{1}\left(t,i\right),\ldots ,x_{M}\left(t,i\right)\right]}^{T}$ represents the M×1 vector of PM-based waveforms during the *i*-th radar scan. $x_{m}\left(t,i\right)$ is the waveform transmitted from the *m*-th transmit element, and n(t,i) is an N×1 vector of zero-mean white Gaussian noise.

The target-scattering signal components associated with the individual PM-based FH waveforms are achieved by using a matched filter bank. As a result, the target scattering signals observed at the output of the MIMO radar receiver (5) are matched-filtered to the proposed PM-based sequences (3), yielding the MN×1 vector of the virtual signal. The extended vector can be expressed as:

(6) $$\begin{array}{ll} r\left(i\right) & =vec\left(\int_{T_{0}}y\left(t,i\right)x^{H}\left(t\right)dt\right)\\ & \;=vec\left(H^{T}\right)+\hat{n}\left(i\right) \end{array}$$

In (6), vec(⋅) describes the vectorization operator that stacks the columns of a matrix into one long column vector, and $\hat{n}\left(i\right)$ denotes the MN×1 zero-mean white Gaussian noise with covariance $\delta_{z}^{2}I_{MN}$.

### 2.2. Information-Embedding Scheme

It is assumed that a phase symbol denotes B bits of binary sequence. During the *i*-th radar pulse, the binary sequence that needs to be embedded is used to select phase symbols $\Omega_{m,q}\left(i\right),m=1,\ldots ,M,q=1,\ldots ,Q$ from a pre-defined constellation of $K=2^{B}$ symbols. It is considered that the constellation is uniformly distributed between [0,2π], which can be expressed as:

(7) $${\mathbb{C}}_{PSK}=\left\{0,\frac{2\pi }{K},\ldots ,\frac{\left(K-1\right)2\pi }{K}\right\}$$

The set of PM-based FH waveforms at time *i* can be rewritten as:

(8) $$x_{m}\left(t,i\right)=\sum_{q=1}^{Q}e^{j\Omega_{m,q}\left(i\right)}e^{j2\pi c_{m,q}\Delta ft}u\left(t-\Delta t\right),m=1,\ldots ,M$$

In (8), $\Omega_{m,q}\left(i\right)\in {\mathbb{C}}_{PSK}$. To simplify discussion and implementation, it is assumed that a communication receiver equipped with a single antenna is located at a known direction $\theta_{c}$. Therefore, the received signal at the output of the communication receiver can be expressed as:

(9) $$y\left(t,i\right)=\alpha_{c}a^{T}\left(\theta_{c}\right)x\left(t,i\right)+n\left(t,i\right)$$

where $\alpha_{c}$ denotes the channel attenuation coefficient. It is considered that $\alpha_{c}$ keeps constant during the whole processing interval. $a\left(\theta_{c}\right)$ indicates the steering vector of the transmit antenna array, and n(t,i) denotes the zero-mean white Gaussian noise with covariance $\delta_{w}^{2}$.

It is assumed that the communication receiver has full knowledge of the code $c_{m,q}$ and the step Δf. Hence, the communication received signals (5) are matched-filtered to the orthogonal FH sub-pulses yields:

(10) $$\begin{array}{ll} r_{m,q}\left(i\right) & =\int_{0}^{\Delta t}y\left(t,i\right)e^{-j2\pi c_{m,q}\Delta ft}u\left(t-\Delta t\right)dt\\ & =\alpha_{c}e^{-j\pi d_{m}\sin\theta_{c}}e^{\Omega_{m,q}\left(\tau \right)}+n_{m,q}\left(i\right)\\ & \;\;m=1,\ldots ,M,q=1,\ldots ,Q \end{array}$$

In (10), $d_{m}$ denotes the distance between the first and the *m*-th antennas of the transmit array. The communication receiver has the ability to undo $e^{-j\pi d_{m}\sin\theta_{c}}$ before it estimates the symbol $\Omega_{m,q}\left(i\right)$. $r_{m,q}\left(i\right)$ is viewed as a phase-rotated and noisy version of the *m*-th entry of $a\left(\theta_{c}\right)$. Hence, $\Omega_{m,q}\left(i\right)$ that need to be embedded is estimated as:

(11) $${\hat{\Omega }}_{m,q}\left(i\right)=Angle\left(r_{m,q}\left(i\right)\right)-Angle\left(\alpha_{c}\right)+2\pi d_{m}\sin\theta_{c}$$

Then, the communication receiver compares ${\hat{\Omega }}_{m,q}\left(i\right)$ to the predefined ${\mathbb{C}}_{PSK}$. $\Omega_{m,q}\left(i\right)$ is restored from $r_{m,q}\left(i\right)$ at the output of the (*m,q*)-th matched filter. It allows the communication receiver to determine $\Omega_{m,q}\left(i\right)$ and convert $\Omega_{m,q}\left(i\right)$ into the original sequence. The advantages of the communication information embedding scheme have been presented in [44].

## 3. Multiple-Input Multiple-Output (MIMO) Radar Ambiguity Function

In this section, we consider a radar target at χ(τ,v,f) where τ describes the delay of the target range, v denotes the Doppler frequency, $f=2\pi \frac{d_{R}}{\lambda }\sin\theta$ indicates the spatial frequency. Here θ and λ are the angle of the target and the wavelength, respectively. $d_{R}$ and $d_{T}$ are the distance between the transmit antennas and between the receiver antennas, respectively. To simplify the discussion, we assume $d_{T}=d_{R}$ in this paper. $x_{m}\left(t\right),m=1,\ldots ,M$ denotes the waveform radiated by the *m*-th transmit antenna. In [17], the MIMO radar ambiguity function can be defined as:

(12) $$\chi \left(\tau ,v,f,f^{\prime }\right)=\sum_{m=1}^{M}\sum_{m^{\prime }=1}^{M}\chi_{m,m^{\prime }}\left(\tau ,v\right)e^{j2\pi \left(fm-f^{\prime }m^{\prime }\right)}$$

where

(13) $$\chi_{m,m^{\prime }}\left(\tau ,v\right)=\int_{-\infty }^{\infty }x_{m}\left(t\right)x_{m^{\prime }}^{*}\left(t+\tau \right)e^{j2\pi vt}dt$$

In (13), $\chi_{m,m^{\prime }}\left(\tau ,v\right)$ describes the cross ambiguity function, which implicates two radar waveforms $x_{m}\left(t\right)$ and $x_{m^{\prime }}^{}\left(t\right)$. $\chi_{m,m^{\prime }}\left(\tau ,v\right)$ is analogous to the SIMO ambiguity function presented in [19]. We now discuss the MIMO radar ambiguity function for the case when $x_{m}\left(t\right)$ is composed of the shifted forms of a rectangular pulse $u_{m}\left(t\right)$.

(14) $$x_{m}\left(t\right)=\sum_{l=1}^{L}u_{m}\left(t-T_{l}\right)$$

where l denotes the number of the rectangular pulse and T describes pulse repetition interval (PRI). The cross ambiguity function of the waveform $x_{m}\left(t\right)$ is defined as:

(15) $$\chi_{m,m^{\prime }}\left(\tau ,v\right)=\sum_{l^{\prime }=1}^{L}\sum_{l=1}^{L}\chi_{m,m^{\prime }}^{u}\left(\tau +T_{l}-T_{l^{\prime }},v\right)e^{j2\pi vTl}$$

where $\chi_{m,m^{\prime }}^{u}\left(\tau ,v\right)$ describes the cross ambiguity function of the rectangular pulses $u_{m}\left(t\right)$ and $u_{m^{\prime }}\left(t\right)$. We assume that the pulse duration $T_{0}$ and the Doppler frequency v are small enough so that $T_{0}v\approx 0$. At a result, the envelope of the Doppler frequency keeps unchanged within an entire pulse period. The cross ambiguity function $\chi_{m,m^{\prime }}^{u}\left(\tau ,v\right)$ reduces to the cross correlation function $r_{m,m^{\prime }}^{u}\left(\tau \right)$, which is not a function of v any more, we obtain:

(16) $$\chi_{m,m^{\prime }}^{u}\left(\tau ,v\right)\approx r_{m,m^{\prime }}^{u}\left(\tau \right)$$

We further assume that little reflections take place at these second trip ranges. Therefore, the cross ambiguity function of $x_{m}\left(t\right)$ can be rewritten as:

(17) $$\chi_{m,m^{\prime }}\left(\tau ,v\right)≃r_{m,m^{\prime }}^{u}\left(\tau \right)\sum_{l=1}^{L}e^{j2\pi vT_{l}}$$

The MIMO ambiguity function of $x_{m}\left(t\right)$ can be expressed as:

(18) $$\chi \left(\tau ,v,f,f^{\prime }\right)=\sum_{m=1}^{M}\sum_{m^{\prime }=1}^{M}r_{m,m^{\prime }}^{u}\left(\tau \right)e^{j2\pi \left(fm-f^{\prime }m^{\prime }\right)}\sum_{l=1}^{L}e^{j2\pi vT_{l}}$$

It is worth noting that the MIMO ambiguity function $\chi \left(\tau ,v,f,f^{\prime }\right)$ depends on the cross correlation functions $r_{m,m^{\prime }}^{u}\left(\tau \right)$. Furthermore, the shifted forms of a rectangular pulse $\left\{u_{m}\left(t\right)\right\}$ just have an impact on the range and spatial resolution. The pulses have no effect on the Doppler resolution. Consequently, to gain a sharp MIMO ambiguity function, these waveforms should be designed such that the function $\Omega \left(\tau ,f,f^{\prime }\right)$ is denoted as:

(19) $$\Omega \left(\tau ,f,f^{\prime }\right)=\sum_{m=1}^{M}\sum_{m^{\prime }=1}^{M}r_{m,m^{\prime }}^{u}\left(\tau \right)e^{j2\pi \left(fm-f^{\prime }m^{\prime }\right)}$$

Next, we discuss the MIMO radar ambiguity function of the PM-based information embedding FH waveforms. The proposed waveforms can be expressed as (3). We intend to obtain the expression for the function $\Omega \left(\tau ,f,f^{\prime }\right)$ in terms of $\left\{c_{m,q}\right\}$ and $\left\{\Omega_{m,q}\right\}$. To derive $\Omega \left(\tau ,f,f^{\prime }\right)$, we therefore begin with the cross correlation function of the PM-based FH waveform. Making use of (3) and (16), the cross correlation function can be expressed as:

(20) $$r_{m,m^{\prime }}^{x}\left(\tau \right)=\sum_{q=1}^{Q}\sum_{q^{\prime }=1}^{Q}\chi^{rect}\left(\tau -\left(q^{\prime }-q\right)\Delta t,\left(c_{m,q}-c_{m^{\prime },q^{\prime }}\right)\Delta f\right)e^{j\Omega_{m,q}}e^{j2\pi \Delta f\left(c_{m,q}-c_{m^{\prime },q^{\prime }}\right)q\Delta t}e^{j2\pi \Delta fc_{m^{\prime },q^{\prime }}\tau }$$

and the function $\Omega \left(\tau ,f,f^{\prime }\right)$ can be expressed as:

(21) $$\Omega \left(\tau ,f,f^{\prime }\right)=\sum_{m,m^{\prime }=1}^{M}\sum_{q,q^{\prime }=1}^{Q}\chi^{rect}\left(\tau -\left(q^{\prime }-q\right)\Delta t,\left(c_{m,q}-c_{m^{\prime },q^{\prime }}\right)\Delta f\right)e^{j\Omega_{m,q}}e^{j2\pi \Delta f\left(c_{m,q}-c_{m^{\prime },q^{\prime }}\right)q\Delta t}e^{j2\pi \Delta fc_{m^{\prime },q^{\prime }}\tau }e^{j2\pi \left(fm-f^{\prime }m^{\prime }\right)}$$

where $\chi^{rect}\left(\tau ,v\right)$ describes the ambiguity function of u(t), which can be denoted by:

(22) $$\chi^{rect}\left(\tau ,v\right)=\{\begin{array}{ll} \frac{\Delta t-\left|\tau \right|}{\Delta t}\sin c\left(v\left(\Delta t-\left|\tau \right|\right)\right)e^{j\pi v\left(\tau +\Delta t\right)}, & \;\mathrm{if}\left|\tau \right|<\Delta t\\ 0, & \;\mathrm{otherwise} \end{array}$$

For M=1, the function is the special case of the SIMO radar. For the general case of the MIMO radar M>1, not only the auto-correlation functions but also the cross-correlation functions between the waveforms should be taken into account such that the function (22) is sharp around $\left\{\left(\tau ,f,f^{\prime }\right)|\tau =0,f=f^{\prime }\right\}$.

## 4. Waveform Optimization

In this section, we aim to enhance the target detection and feature estimation performance by maximizing the MI between the target response and the target returns in the first step, and then minimizing the MI between successive target scattering signals in the second step. These two stages correspond to the design of the ensemble of excitations and the selection of a suitable signal out of the ensemble, respectively. The two-step cognitive waveform design strategy is based upon continuous learning from the radar scene. The dynamic information about the target feature is utilized to design PM-based FH codes. In this way the transmitter adjusts its probing signals to suit the dynamically changing environment.

Step 1: this step involves the design of PM-based FH waveforms for the dual-function transmit array. The main idea of waveform design is to maximize the MI between the target scattering signal and the target response, subject to the transmit power constraint.

**Figure 2:**
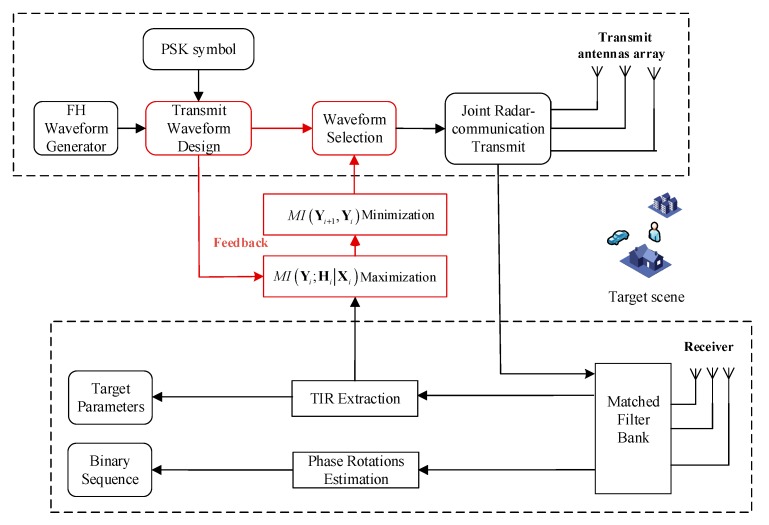
The architecture of an adaptive dual-function MIMO radar communication system.

Step 2: once an ensemble of optimal transmit waveforms has been designed, then we select the most reasonable waveform for emission from the ensemble. The key concept of waveform selection is to minimize the MI between the target scattering signals at present and the next target-scattering signals. The step ensures that we continually obtain target scattering signals that are independent of each other in time, in order to achieve more feature information of the target at each time instant of reception. Figure 2 describes the architecture of an adaptive dual-function MIMO radar communication system.

Step 1: during the i-th radar scan, the set of PM-based FH waveforms $x_{m}\left(t,i\right),m=1,\ldots ,M$ can be expressed as a matrix $X_{i}\in {\mathbb{C}}^{M\times K}$ after discrete sampling, where ℂ describes the number domain and K indicates the sample number. $N\in {\mathbb{C}}^{N\times K}$ is zero-mean white noise matrix. Therefore, the N×K matric of the target scattering signals is written as:

(23) $$Y_{i}=H_{i}^{T}X_{i}+N$$

Here $H_{i}∼\mathbb{C}Ν\left(0,R_{H_{i}}\right)$ and $N∼\mathbb{C}Ν\left(0,R_{N}\right)$, and $R_{H_{i}}=E\left\{H_{i}H_{i}^{H}\right\}$ and $R_{N}=E\left\{N^{H}N\right\}$ indicate the covariance matrices of the channel response $H_{i}$ and the zero-mean white noise N, respectively. We intend to maximize the MI between the target-scattering signal and the target response given the transmit waveforms. This involves that the target-scattering signals would be more dependent upon the actual target feature information. According to the definition of MI, we have:

(24) $$I\left(Y_{i};H_{i}|X_{i}\right)=H\left(Y_{i}|X_{i}\right)-H\left(N\right)$$

In (24), $I\left(Y_{i};H_{i}|X_{i}\right)$ represents the MI between two random variates $Y_{i}$ and $H_{i}$ given the transmit matrix $X_{i}$, and $H\left(Y_{i}|X_{i}\right)$ indicates the conditional entropy that $X_{i}$ conveys about $Y_{i}$. The main objective of this step is to maximize $I\left(Y_{i};H_{i}|X_{i}\right)$ between $Y_{i}$ and $H_{i}$ given $X_{i}$. According to the definition of entropy, we have:

(25) $$H\left(Y_{i}|X_{i}\right)=\int -p\left(Y_{i}|X_{i}\right)In\left[p\left(Y_{i}|X_{i}\right)\right]dY_{i}$$

Here $p\left(Y_{i}|X_{i}\right)$ indicates the conditional probability density function (PDF) of the received matrix $Y_{i}$ given transmit matrix $X_{i}$. The conditional PDF $p\left(Y_{i}|X_{i}\right)$ can be expressed as follows:

(26) $$\begin{array}{ll} p\left(Y_{i}|X_{i}\right) & =\prod_{n=1}^{N}p\left(y_{n,i}|X_{i}\right)\\ & =\prod_{n=1}^{N}\frac{1}{\pi^{K}\det\left(X_{i}^{H}R_{H_{i}}X_{i}+R_{N}\right)}e^{-y_{n,i}^{H}{\left(X_{i}^{H}R_{H_{i}}X_{i}+R_{N}\right)}^{-1}y_{n,i}}\\ & \;=\frac{1}{\pi^{NK}{\left[\det\left(X_{i}^{H}R_{H_{i}}X_{i}+R_{N}\right)\right]}^{N}}e^{-tr\left[{\left(X_{i}^{H}R_{H_{i}}X_{i}+R_{N}\right)}^{-1}Y_{i}^{H}Y_{i}\right]} \end{array}$$

Solving (25) and (26) gives rise to the following result for the conditional entropy [40]:

(27) $$H\left(Y_{i}|X_{i}\right)=NKIn\left(\pi \right)+NK+NIn\left[\det\left(X_{i}^{H}R_{H_{i}}X_{i}+R_{N}\right)\right]$$

Similarly, the result for the entropy of the noise can be derived as follows:

(28) $$H\left(N\right)=NKIn\left(\pi \right)+NK+NIn\left[\det\left(R_{N}\right)\right]$$

Making use of Equations (24), (27) and (28), the MI between the target-scattering signal and the channel response given transmit waveforms can be rewritten as follows:

(29) $$I\left(Y_{i};H_{i}|X_{i}\right)=NIn\left[\det\left(X_{i}^{H}R_{H_{i}}X_{i}+R_{N}\right)\right]-NIn\left[\det\left(R_{N}\right)\right]$$

Therefore, we can formulate the MI maximization criterion as follows:

(30) $$\begin{array}{l} \underset{X_{i}}{\max}\left\{NIn\left[\det\left(X_{i}^{H}R_{H_{i}}X_{i}+R_{N}\right)\right]-NIn\left[\det\left(R_{N}\right)\right]\right\}\frac{n!}{r!\left(n-r\right)!}\\ s.t.\;\mathrm{tr}\left[X_{i}^{H}X_{i}\right]\leq P_{0}\\ \;\;\;\;\;c_{m,q}\neq c_{m^{\prime },q},\;\mathrm{for}\;m=m^{\prime },\forall q\;\;\;\Delta t\Delta f=1 \end{array}$$

where $P_{0}$ indicates the transmit power. The work of [40] has given a rigorous solution of the above optimization problem (30). Then, we can obtain the ensemble ${\mathbb{C}}_{{\hat{X}}_{i}}$ out of the whole set of PM-based FH sequences, and the corresponding power allocation vector over diverse dual-function transmit antennas. We start the design procedure with the transmit waveforms from the PM-based FH matrix, and allocate the power on a pulse level as well as across the transmit antennas based on the MI maximization criterion.

Step 2: we then proceed to the waveform selection procedure, in which the successive target scattering signals are different from each other. The step ensures that we achieve more information of the target feature at each time instant of reception. We denote the MI between the successive target scattering signals at time i and at time i+1 as:

(31) $$I\left(Y_{i+1},Y_{i}\right)=H\left(Y_{i+1}|X_{i+1}\right)+H\left(Y_{i}|X_{i}\right)-H\left(Y_{i+1},Y_{i}|X_{i+1},X_{i}\right)$$

In (31), the term $H\left(Y_{i}|X_{i}\right)$ (or $H\left(Y_{i+1}|X_{i+1}\right)$) denotes the entropy of $Y_{i}$ (or $Y_{i+1}$) at time i (or i+1) given the knowledge of $X_{i}$ (or $X_{i+1}$). The term $H\left(Y_{i+1},Y_{i}|X_{i+1},X_{i}\right)$ is defined similarly. According to the literature [6,34], the above Equation (31) can be rewritten as follows:

(32) $$\begin{array}{ll} I\left(Y_{i},Y_{i+1}\right) & =-NIn\left(\det\left(I_{\left(M\times M\right)}-D_{i,i+1}^{2}\right)\right)\\ & =-N\sum_{m=1}^{M}In\left(1-{\left(d_{i,i+1}^{m}\right)}^{2}\right) \end{array}$$

where $d_{i,i+1}^{m}(d_{i,i+1}^{1}\geq d_{i,i+1}^{2}\geq \ldots \geq d_{i,i+1}^{M})$ is the diagonal element of the diagonal matrix $D_{i,i+1}^{}$. $D_{i,i+1}^{}$ is achieved by singular value decomposition (SVD) of the covariance matrix, which can be denoted as:

(33) $$R_{Y_{i},Y_{i+1}}=E\left\{Y_{i}^{H}Y_{i+1}\right\}=X_{i}^{H}R_{H_{i}}^{}X_{i+1}$$

In (33), $R_{Y_{i},Y_{i+1}}$ is the cross-covariance of the expressions for $Y_{i}^{}$ and $Y_{i+1}$. Hence, the MI minimization criterion can be expressed as:

(34) $$\begin{array}{l} \underset{X_{i+1}\in {\mathbb{C}}_{{\hat{X}}_{i}}}{\min}\left\{-N\sum_{m=1}^{M}In\left\{1-{\left(d_{i,i+1}^{m}\right)}^{2}\right\}\right\}\\ s.t.\;\mathrm{tr}\left[X_{i+1}^{H}X_{i+1}\right]\leq P_{0}\\ c_{m,q}\neq c_{m^{\prime },q},\;\;\mathrm{for}\;m=m^{\prime },\forall q\;\;\Delta t\Delta f=1 \end{array}$$

We can estimate the values for $Y_{i+1}$ over all possible values of ${\hat{X}}_{i+1}\in {\mathbb{C}}_{{\hat{X}}_{i}}$ using (23). Thus, we can also form an estimate of all the values of the corresponding $D_{i,i+1}^{}$ $\left(d_{i,i+1}^{m}(d_{i,i+1}^{1}\geq d_{i,i+1}^{2}\geq \ldots \geq d_{i,i+1}^{M})\right)$ and choose the value for ${\hat{X}}_{i+1}$ that minimizes (34). The above waveform optimization problem (34) is convex. We can obtain the optimal solution directly by using a MATLAB optimization toolbox, such as CVX.

Step 1 designs the optimal ensemble ${\mathbb{C}}_{{\hat{X}}_{i}}$ based on MI maximization criterion over the spatial domain, and step 2 selects the optimized PM-based FH waveform for each dual-functional transmit antenna from the ensemble ${\mathbb{C}}_{{\hat{X}}_{i}}$ based on MI minimization criterion over the temporal domain. The proposed adaptive waveform design and selection procedures can be summarized as Algorithm 1.
**Algorithm 1**. The adaptive waveform design and selection algorithmInitialize the transmit matrix $X_{0}$ and the covariance matrix of the noise $R_{N}^{}$. 1. At the initial time t=0, measure the target scattering signal $Y_{0}$ and calculate the covariance of the target-scattering signal $R_{Y_{0}}$. The covariance matrix of the channel response $R_{H_{0}}^{}$ can be obtained through successive measurements with uniform power allocation over the transmit antennas by solving (36). 2. The optimized waveform ensemble ${\mathbb{C}}_{{\hat{X}}_{0}}$ can be obtained through the waveform design process by solving (30). 3. At time t=1, measure $Y_{1}$ and calculate $R_{Y_{1}}$ and the cross-covariance matrix $R_{Y_{0},Y_{1}}$. The actual channel response $H_{1}$ is obtained by de-convolving the target-scattering signal with the transmit signal by using (23). 4. Calculate the corresponding singular values $d_{0,1}^{m},m=1,\ldots ,M$ of $R_{Y_{0},Y_{1}}$ and ${\hat{X}}_{1}\in {\mathbb{C}}_{{\hat{X}}_{0}}$ can be acquired through the waveform selection process by solving (38). 5. At time t=1, emission ${\hat{X}}_{1}$ and observe the corresponding received signal ${\hat{Y}}_{1}$ to achieve $R_{H_{1}}^{}$ by using (36). 6. If $i=I_{\max}$, the iterative procedure ends; or else, repeat steps 2–5 iteratively.

**Figure 3:**
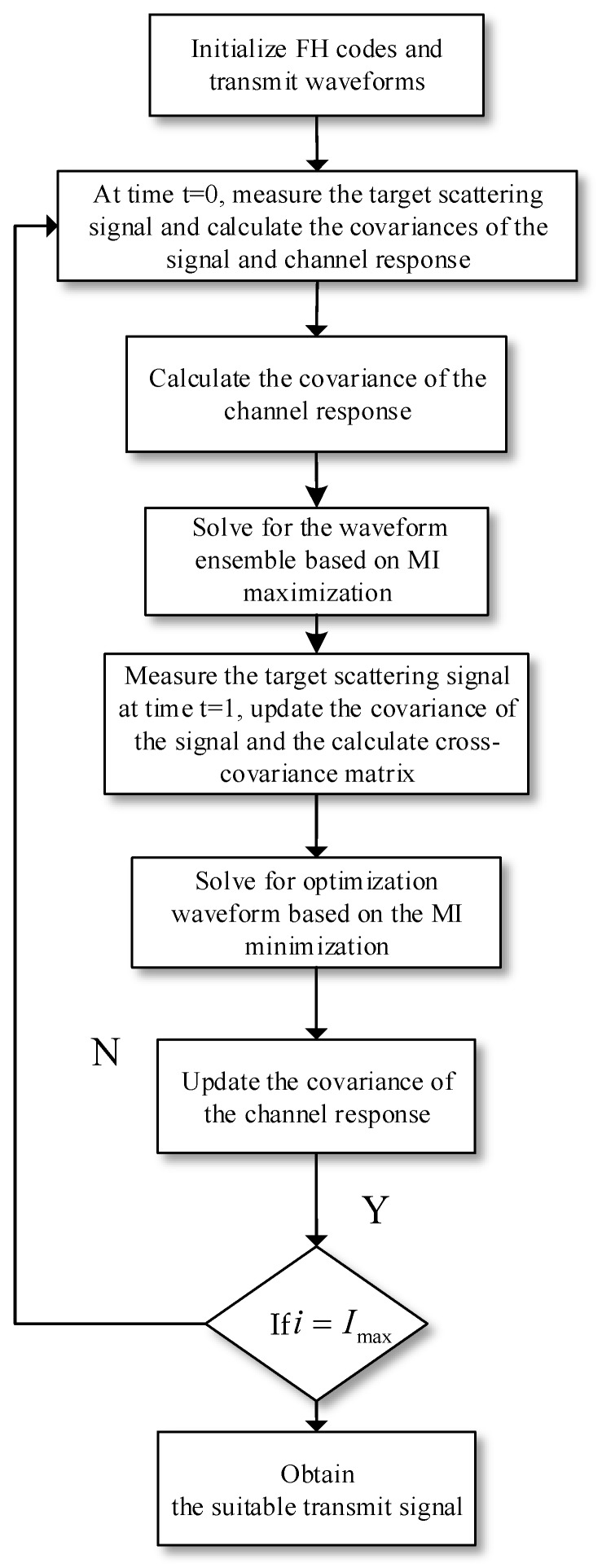
The adaptive waveform design and selection scheme for target detection.

It is worth noting that adaptation is included in the waveform design and selection procedures through the feedback process and numerous interactions with the radar channel. In summary, the adaptive waveform optimization strategy for target detection is implemented according to the block diagram in Figure 3.

## 5. Simulation

In this section, numerical results based on Monte Carlo simulations have been provided to validate the effectiveness of the proposed method. Without loss of generality, each entry of the channel matrices follows the standard complex Gaussian distribution. The simulation parameters are based on radar application with a high Pulse Repetition Frequency (PRF), such as in X-band radar. A data rate in the range of dozens of Mbps can be achieved. We provide a comparison between the proposed scheme and the method of [32]. To implement the method in [32], we consider a dual-function MIMO system operating in the X-band with carrier frequency $f_{c}=8.2$ GHz and bandwidth B=500 MHz. The sampling frequency is $f_{s}=10^{9}$ sample/sec, which is taken as the Nyquist rate. The PRI is $T_{0}=10\;\mu s$. We assume an arbitrary linear transmit array consisting of M=16 elements. We further assume that the minimum transmit/receive antenna spacing is sufficiently larger than half wavelength (distributed MIMO configuration). Hence, the correlation introduced by finite antenna element spacing is low enough that the fades associated with two different antenna elements can be considered independent. To implement the radar function, we further assume that the FH step is Δf=10 MHz, the length of the FH code is Q=20 and the FH interval duration is Δt=0.1 μs. We generate a set of 16 FH pulse waveforms. The parameter J=50 is used. Therefore, the 320 FH code is generated randomly from the set {1,2,…,J}, where J=50.

We employ orthogonal sequences of the FH pulse over the transmit antenna elements. The backscatter signals are matched filtered at the receivers and the transmitted signals are later modified by the waveform optimization module as shown in Figure 2. The optimized transmission sequence at one particular transmit antenna after the two-step optimization process. The target response extracted from the received target echoes after matched filtering at the end of 20 iterations of the algorithm, where an excellent performance of the target response estimation can be observed. At each iteration, the scattering coefficients for the target and non-target scatterers in $H_{i}$ vary as described by the Swerling III model [45,46]. This causes the amplitude returns of the backscatter signals to vary at each instance. However, the amplitudes of the echoes from the target are always assumed to be stronger than those from the clutter sources. 

### 5.1. Target Detection Performance

**Figure 4:**
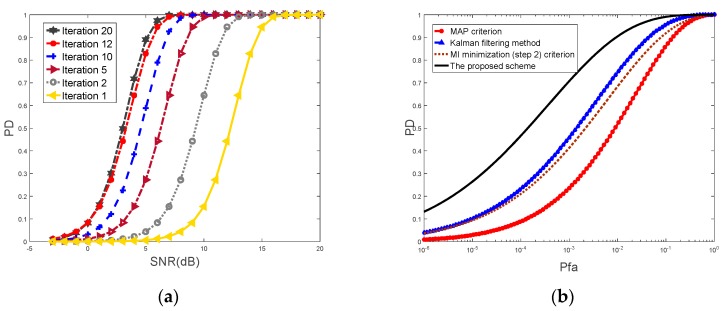
(**a**) The detection probability for different iterations provided by the proposed scheme; (**b**) The receiver operating characteristics (ROC) for four approaches.

Figure 4a illustrates the detection probability offered by the proposed scheme versus the signal-to-noise ratio (SNR) for different iterations. The iteration process is run 20 times. All optimal waveforms are generated by the proposed two-step algorithm. The value of requested SNR increases as the probability increase for a particular number of iterations. The SNR decreases as the number of iterations increase for a certain detection probability. The detection performance offered by the proposed scheme improves as the number of iterations increases. Simulation results show that, on average, 20 iterations of the waveform optimization algorithm are required in order to achieve convergence of the target response estimation for a wide range of radar scenes.

Figure 4b illustrates the ROC for the following four approaches while the value of received SNR equals to 8 dB. (1) 4×4 MIMO system based on conventional maximum a posterior (MAP) approach; (2) 4×4 MIMO system using the Kalman filtering [42]; (3) 4×4 MIMO system based on MI optimization (step 2) scheme; (4) 4×4 dual-function MIMO radar-communications system using the proposed scheme. 

The plot is run at the end of 24 iterations. For the probability of false alarm $P_{fa}=0.005$, the of target detection probability generated by the proposed scheme is 0.95 as compared with 0.75 provided by the Kalman filtering method, 0.7 by MI optimization (step 2) criterion and 0.55 by conventional MAP approach. As the two-step scheme can use the temporal correlation of target characteristic during the radar scan interval, the dual-function MIMO system constantly adapts transmit mode to suit the dynamic radar scene. Furthermore, the successive target returns are considered as independent of each other. The property guarantees that information about the radar scene is learned at each instant of reception. In this case, the detection performance offered by the proposed scheme is best.

### 5.2. Target Response Estimation Performance

**Figure 5:**
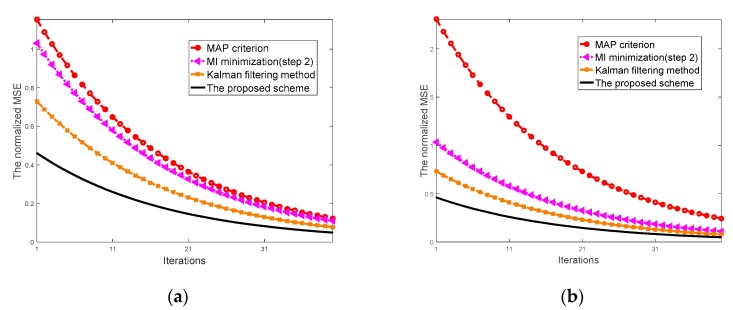
(**a**) The mean-square error (MSE) of target response estimation subject to power constraint; (**b**) the MSE of target response estimation subject to power and probability constraint.

Figure 5a displays the MSE achieved by the proposed scheme with regard to the estimation of target response. This plot demonstrates an improved MSE performance for the two-step optimization as compared with the conventional MAP approach, the Kalman filtering method and the MI optimization (step 2) modules, particularly for the first few iterations.

It is evident from Figure 5 that the MSE performance offered by the proposed scheme is superior to the conventional MAP approach and the Kalman filtering method. The MSE performance offered by the proposed scheme is superior to MI optimization (step 2) criterion. Similarly, Figure 5b shows the MSE performance offered by the proposed scheme with respect to the estimate of target response. The MSE of target response estimation provided by the two-step scheme and other several approaches are compared to reveal the benefit of the proposed scheme.

### 5.3. Delay-Doppler Resolution Performance

**Figure 6:**
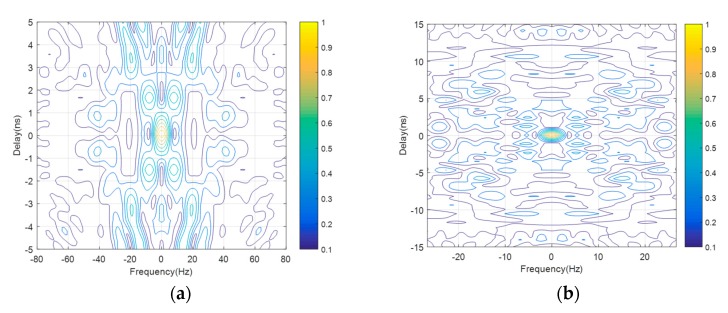
Ambiguity Function (AF) contours showing delay-Doppler resolution at (**a**) iteration 1 and (**b**) iteration 20, which demonstrates smaller focal area in (**b**) as compared with (**a**).

Figure 6a displays the 2×2 MIMO radar ambiguity function contours of the original waveform. The delay-Doppler resolution deteriorates owing to the presence of environment noise as shown in Figure 6a. This phenomenon will became worse if the interference scatterers are placed in the vicinity along the line linking the radar target and the antenna. The radar target is considered to be located at the origin of the plan. Figure 6b displays the MIMO radar ambiguity function contours of the optimization waveform by the proposed scheme at the end of 20 iterations. As can be seen from Figure 6a, the target discrimination capability becomes significantly enhanced by the increasing iteration number. Specifically, the noise is suppressed by about 2.5 dB. 

The delay-Doppler resolution, which is the output of matched filter at the radar receiver, is connected to the MIMO radar ambiguity function of the transmit waveforms. The near-ideal thumbtack response would appear if the statistically independent waveforms are employed for emission. We constantly use the optimization waveforms provided by the proposed scheme to match the estimated target response. It is worth noting that the estimated target response is continuously updated at each iteration. At a result, the matching process ensures that the noise interference can be suppressed over the radar channels and further enhance the SNR of the target-scattering signals. Figure 6b illustrates the enhancement in SNR with multiple iteration number. The phenomenon shows the enhanced ability of the proposed dual-function MIMO system to discriminate the target from radar environment and resolve the target’s range and velocity.

### 5.4. Symbol Error Rate Performance

**Figure 7:**
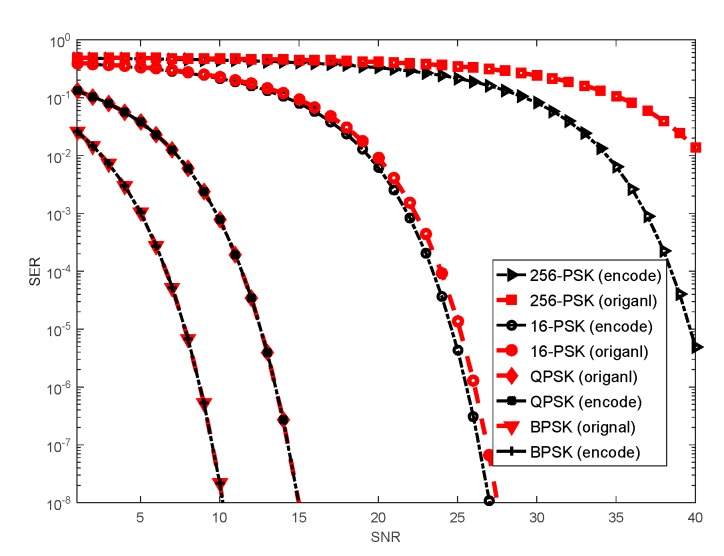
The symbol error rates (SERs) versus signal-to-noise ratio (SNR) for Binary Phase Shift Keying (BPSK), Quadrature Phase Shift Keying (QPSK), 16-Phase Shift Keying (16-PSK) and 256-Phase Shift Keying (256-PSK).

We study the SER performance of the communication source embedding scheme using Binary Phase Shift Keying (BPSK), Quadrature Phase Shift Keying (QPSK), 16-Phase Shift Keying (16-PSK) and 256-Phase Shift Keying (256-PSK) constellations and compare the original waveform with the encoded waveform. The original waveform corresponds to data rate of R = 32; 64; 128 and 256 Mbps, respectively. To obtain encoded waveform, a convolutional encoder of rate 2/3 is employed in the original waveform. We use a Viterbi decoder to decode the received encoded waveform at the communication receiver. The communication channel coefficient is taken as $\left|\alpha_{c}\right|=1$, and the phase of $\alpha_{c}$ is uniformly distributed within the interval [0,2π]. To test the SER performance, we generate a number of $10\times 10^{17}$ random BPSK, QPSK, 16-PSK and 256-PSK symbols. The SERs versus SNR for all constellation sizes is illustrated in Figure 7.

The results demonstrate that the smaller the constellation size is, the better the SER performance will be. As the constellation size increases, it is more difficult to detect the symbols. It can be explained that the defective cross-correlation between the non-orthogonal transmit sequences influences the detection performance. Obviously, the encoded waveform achieves greater SER performance as compared to the original waveform. Therefore, intersymbol interference is a significant source of detection error resulting in performance degradation. It is expected that the SER performance gets worse if longer FH waveforms are used. It is worth noting that, for all techniques tested, the SER performance offered by the encoded waveform outperforms the performance provided by the original waveform. Therefore, the SER superiority comes at the price of lower data rate.

**Figure 8:**
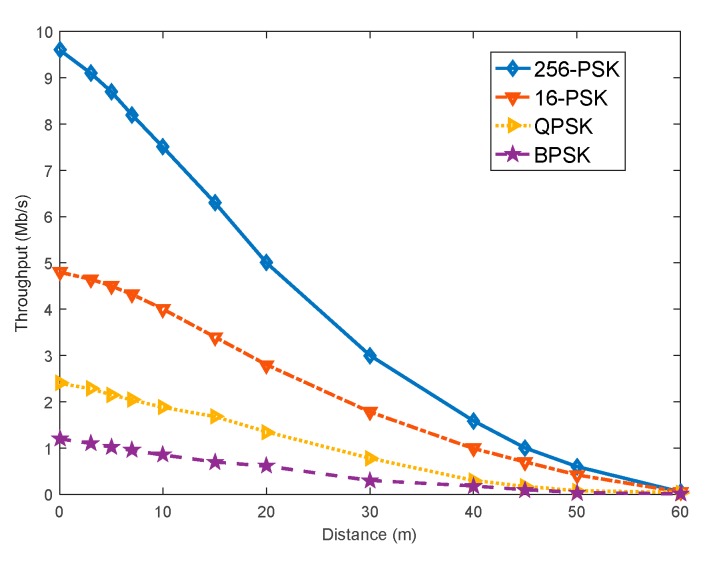
Comparative throughput of BPSK, QPSK, 16-PSK, 256-PSK.

Figure 8 illustrates the throughput result provided by the proposed optimization waveform versus distance for BPSK, QPSK, 16-PSK, and 256-PSK constellation. 256-PSK waveform provides a data rate of approximately 8 Mbps at a distance of 10 m, which is better than that generated by BPSK, QPSK, 16-PSK constellations. The highest data rate is acquired by the 256-PSK waveform within a distance of 60 m, as the distance between the system nodes increases the data rate decreases.

### 5.5. Detection Variation Performance

The detection constraint optimization has recently been studied in works such as [38,39], where the authors address the problem of radar code design for target recognition in the presence of colored Gaussian disturbance. The objective function in [38] aims to maximize the weighted average Euclidean distance between the ideal echoes from different target hypotheses. Furthermore, additional practical constraints are considered in [38]. For example, the modulus of the waveform is restricted to be a constant and the detection constraints require that the achievable SNR for each target hypothesis is larger than a desired threshold.

**Figure 9:**
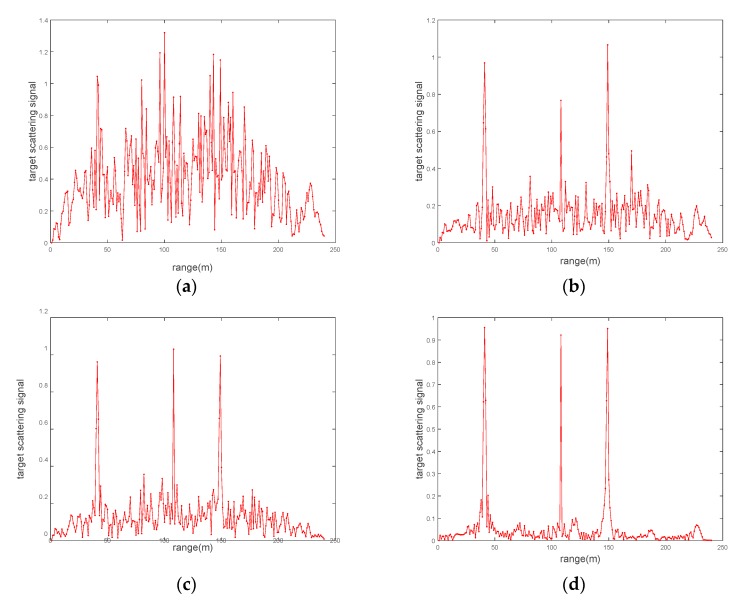
Target-scattering signal profiles at (**a**) iteration 1, (**b**) iteration 5, (**c**) iteration 10, and (**d**) iteration 20.

Figure 9 indicates the detection variation offered by the proposed scheme subject to the detection constraint. We assume a radar scene, which has three range-separated targets. The target-scattering signals derived from the radar scene is normalized and the proposed dual-function MIMO system intends to discriminate the scatterers by using a particular detection threshold.

With subsequent iterations of the proposed algorithm, the detection performance of the multiple targets is enhanced. As can be seen from Figure 9, by suppressing noise, the dual-function MIMO system could discriminate three scatterers effectively at the end of 20 iterations. The result proves the performance of target detection presented in Figure 6b as well.

The detection performance is enhanced by providing the waveform design (step 1) procedure in the proposed scheme. The waveform design procedure ensures the maximum of the Euclidean distance between the target echoes from different scatterers based on the MI maximization criterion in (30). The proposed design procedure is similar to the optimization method in [43]. By increasing the number of iterations, we can obtain more accurate estimates of the target responses, which are used to improve detection of the multiple targets.

### 5.6. Comparison with Other Methods

**Figure 10:**
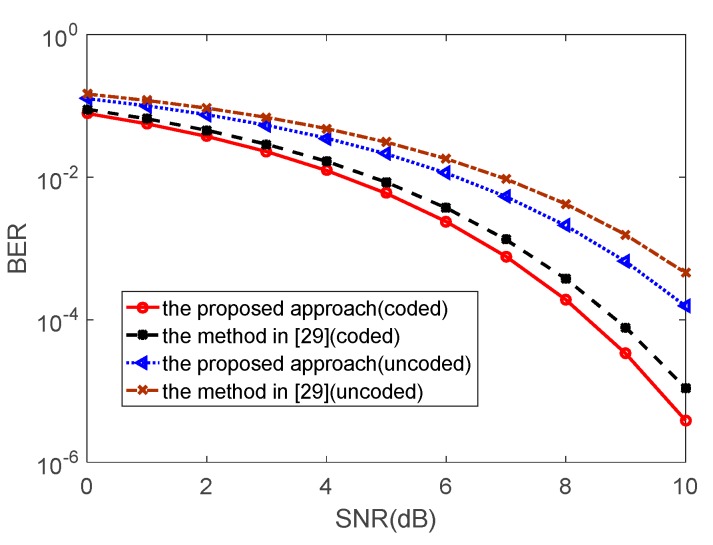
The SERs versus SNR for BPSK.

We have assess the performance of the new approach in terms of the Bit Error Rate (BER) and compare it with the method in [29]. Note that the latter approach uses a single sequence in tandem with 4 sidelobe levels towards the communication direction to deliver 2 bits of information. On the other hand, we use a PM-based FH waveform to deliver two bits per pulse. To test the BER, a sequence of symbols unencoded (two bits each) is used. Furthermore, a convolutional encoder of rate 2/3 is used in the original waveform leading to encoded waveform. Both the unencoded and the encoded waveforms are embedded independently using the approach of [29] and the new approach. The received encoded signal is decoded using a Viterbi decoder. The BERs versus the signal-to-noise ratio (SNR) for the two approaches is presented in Figure 10 for both the unencoded as well as the encoded data sequences. Obviously, the proposed approach achieves better BER performance compared to the method of [29]. Note that the latter approach embeds 25% of the information via each of the four beams. Thus, intersymbol interference is a non-negligible source of detection error leading to capability degradation. The behavior can be expected to be worse if longer pulses are transmitted. Note that, for two approaches tested, the BER with respect to the encoded waveform outperforms the BER with respect to the unencoded waveform. However, this BER superiority comes at the price of slower data transmission rate.

**Figure 11:**
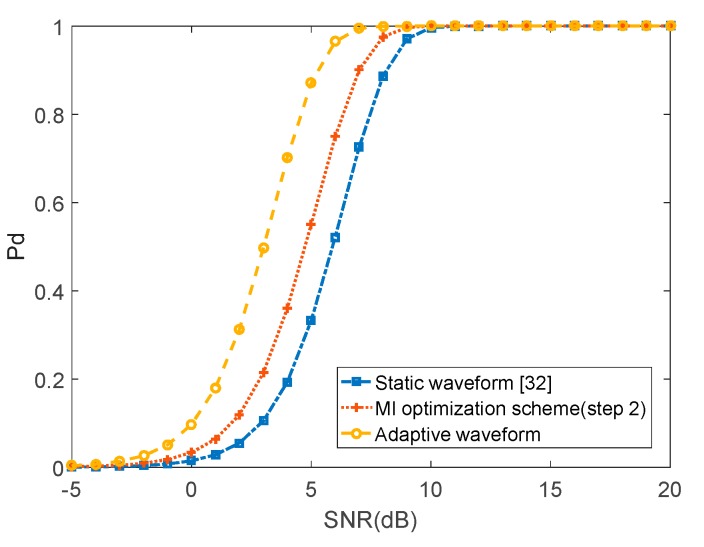
Probability of detection for adaptive waveform and static waveform.

In Figure 11, we compare the performance for adaptive waveform provided by the proposed method to the performance for a static waveform [32] over multiple snapshots. As the proposed method selects specific waveforms, which generate target returns having low correlation over time, the system adapts its transmit waveform better to the fluctuating target Radar Cross-Section (RCS). On the other hand, the static waveform [32] is unable to match the time-varying target response. Therefore, the performance of the static waveform [32] is worse than the proposed adaptive waveform.

## 6. Conclusions

The problem of dual-functionality system design for joint radar and communications operation was considered. We further presented a two-step waveform optimization algorithm for the proposed system, which combines the waveform optimization and selection processes. The proposed algorithm is based upon constant learning of the radar scene at the receiver and adaptation of the probing codes to suit the dynamic target feature. The adaptive process ensures maximum information extraction from the target of interest. The effectiveness of the proposed technique and its superiority over existing techniques in terms of the BER performance and the target detection performance were demonstrated through extensive simulations. The proposed system would form a joint platform for future intelligent transportation applications for which both environmental perception and establishment of data links are crucial. Future research will look into the tradeoff between the performance improvement offered by the proposed approach and the computational complexity involved.

## References

[1] Li J., Stoica P. (2009). MIMO Radar Signal Processing.

[2] Li B., Petropulu A.P., Trappe W. (2016). Optimum co-design for spectum sharing between matrix completion based MIMO radars and a MIMO communication system. IEEE Trans. Signal Process..

[3] Qian J., Lops M., Zheng L., Wang X., He Z. (2018). Joint System Design for Coexistence of MIMO Radar and MIMO Communication. IEEE Trans. Signal Process..

[4] Liu S., Zhang Y.D., Shan T., Tao R. (2018). Structure-Aware Bayesian Compressive Sensing for Frequency-Hopping Spectrum Estimation with Missing Observations. IEEE Trans. Signal Process..

[5] Fan W., Liang J., Li J. (2018). Constant Modulus MIMO Radar Waveform Design with Minimum Peak Sidelobe Transmit Beampattern. IEEE Trans. Signal Process..

[6] Yao Y., Zhao J., Wu L. (2018). Adaptive Waveform Design for MIMO Radar-Communication Transceiver. Sensors.

[7] Wu L., Babu P., Palomar D.P. (2017). Transmit Waveform/Receive Filter Design for MIMO Radar with Multiple Waveform Constraints. IEEE Trans. Signal Process..

[8] Stoica P., Li J., Xie Y. (2007). On Probing Signal Design for MIMO Radar. IEEE Trans. Signal Process..

[9] Tang B., Li J. (2018). Spectrally Constrained MIMO Radar Waveform Design Based on Mutual Information. IEEE Trans. Signal Process..

[10] Yang Y., Blum R. (2007). MIMO radar waveform design based on mutual information and minimum mean-square error estimation. IEEE Trans. Aerosp. Electron. Syst..

[11] Wang L., Zhu W., Zhang Y., Liao Q., Tang J. (2018). Multi-Target Detection and Adaptive Waveform Design for Cognitive MIMO Radar. IEEE Sens. J..

[12] Fuhrmann D.R., San Antonio G. (2009). Transmit beamforming for MIMO radar systems using signal crosscorrelation. IEEE Trans. Aerosp. Electron. Syst..

[13] Song X., Zhou S., Willett P. (2010). Reducing the Waveform Cross Correlation of MIMO Radar with Space–Time Coding. IEEE Trans. Signal Process..

[14] Jajamovich G.H., Lops M., Wang X. (2010). Space-Time Coding for MIMO Radar Detection and Ranging. IEEE Trans. Signal Process..

[15] Cheng Z., Liao B., He Z., Li Y., Li J. (2018). Spectrally Compatible Waveform Design for MIMO Radar in the Presence of Multiple Targets. IEEE Trans. Signal Process..

[16] Yang Y., Blum R.S., He Z.S., Fuhrmann D.R. (2010). MIMO radar waveform design via alternating projection. IEEE Trans. Signal Process..

[17] Antonio G.S., Fuhrmann D.R., Robey F.C. (2007). MIMO Radar Ambiguity Functions. IEEE J. Sel. Top. Signal Process..

[18] Chen C.-Y., Vaidyanathan P. (2008). MIMO Radar Ambiguity Properties and Optimization Using Frequency-Hopping Waveforms. IEEE Trans. Signal Process..

[19] Gogineni S., Nehorai A. (2012). Frequency-Hopping Code Design for MIMO Radar Estimation Using Sparse Modeling. IEEE Trans. Signal Process..

[20] Han K., Nehorai A. (2016). Jointly optimal design for mimo radar frequency-hopping waveforms using game theory. IEEE Trans. Aerosp. Electron. Syst..

[21] Griffiths H., Cohen L., Watts S., Mokole E., Baker C., Wicks M., Blunt S. (2015). Radar spectrum engineering and management: Technical and regulatory issues. Proc. IEEE.

[22] Chiriyath A.R., Paul B., Jacyna G.M., Bliss D.W. (2015). Inner Bounds on Performance of Radar and Communications Co-Existence. IEEE Trans. Signal Process..

[23] Ahmed A., Zhang Y.D., Gu Y. (2018). Dual-function radar-communications using QAM-based sidelobe modulation. Digit. Signal Process..

[24] Bliss D.W. (2014). Cooperative radar and communications signaling: The estimation and information theory odd couple. Proceedings of the 2014 IEEE Radar Conference, Cincinnati, OH, USA, 19–23 May 2014.

[25] Geng Z., Deng H., Himed B. (2015). Adaptive radar beamforming for interference mitigation in radar-wireless spectrum sharing. IEEE Signal Process. Lett..

[26] Junhui Z., Tao Y., Yi G., Jiao W., Lei F. (2013). Power control algorithm of cognitive radio based on non-cooperative game theory. China Commun..

[27] Khawar A., Abdelhadi A., Clancy C., Clancy T. (2015). Target Detection Performance of Spectrum Sharing MIMO Radars. IEEE Sens. J..

[28] Ahmed A., Zhang Y.D., Himed B. (2019). Distributed Dual-Function Radar-Communication MIMO System with Optimized Resource Allocation. Proceedings of the 2019 IEEE Radar Conference (RadarConf), Boston, MA, USA, 22–26 April 2019.

[29] Hassanien A., Amin M.G., Zhang Y.D., Ahmad F. (2016). Dual-function radar-communications: Information embedding using sidelobe control and waveform diversity. IEEE Trans. Signal Process..

[30] Hassanien A., Amin M.G., Zhang Y.D., Ahmad F. (2016). Signaling strategies for dual-function radar communications: An overview. IEEE Aerosp. Electron. Syst. Mag..

[31] Hassanien A., Amin M.G., Zhang Y.D., Ahmad F. (2016). Phase-modulation based dual-function radar-communications. IET Radar, Sonar Navig..

[32] Hassanien A., Himed B., Rigling B.D. (2017). A dual-function MIMO radar-communications system using frequency-hopping waveforms. Proceedings of the 2017 IEEE Radar Conference (RadarConf), Seattle, WA, USA, 8–12 May 2017.

[33] Ahmed A., Gu Y., Silage D., Zhang Y.D. (2018). Power-Efficient Multi-User Dual-Function Radar-Communications. Proceedings of the 2018 IEEE 19th International Workshop on Signal Processing Advances in Wireless Communications (SPAWC), Kalamata, Greece, 25–28 June 2018.

[34] Yao Y., Zhao J., Wu L. (2018). Cognitive Radar Waveform Optimization Based on Mutual Information and Kalman filtering. Entropy.

[35] Zhao J., Guan X., Li X.P. (2013). Power allocation based on genetic simulated annealing algorithm in cognitive radio networks. Chin. J. Electron..

[36] Bell M. (1993). Information theory and radar waveform design. IEEE Trans. Inf. Theory.

[37] Romero R.A., Bae J., Goodman N.A. (2011). Theory and Application of SNR and Mutual Information Matched Illumination Waveforms. IEEE Trans. Aerosp. Electron. Syst..

[38] Tang B., Tang J., Peng Y. (2010). MIMO Radar Waveform Design in Colored Noise Based on Information Theory. IEEE Trans. Signal Process..

[39] Aubry A., De Maio A., Huang Y., Piezzo M., Farina A. (2015). A new radar waveform design algorithm with improved feasibility for spectral coexistence. IEEE Trans. Aerosp. Electron. Syst..

[40] Chen Y., Nijsure Y., Yuen C., Chew Y.H., Ding Z., Boussakta S. (2013). Adaptive Distributed MIMO Radar Waveform Optimization Based on Mutual Information. IEEE Trans. Aerosp. Electron. Syst..

[41] Naghibi T., Behnia F. (2011). MIMO Radar Waveform Design in the Presence of Clutter. IEEE Trans. Aerosp. Electron. Syst..

[42] Yao Y., Zhao J., Wu L. (2018). Waveform Optimization for Target Estimation by Cognitive Radar with Multiple Antennas. Sensors.

[43] Wang X., Hassanien A., Amin M.G. (2019). Dual-Function MIMO Radar Communications System Design Via Sparse Array Optimization. IEEE Trans. Aerosp. Electron. Syst..

[44] Yao Y., Zhao J., Wu L. (2019). Frequency-Hopping Code Design for Target Detection via Optimization Theory. J. Optim. Theory Appl..

[45] Nijsure Y., Chen Y., Boussakta S., Yuen C., Chew Y.H., Ding Z. (2012). Novel System Architecture and Waveform Design for Cognitive Radar Radio Networks. IEEE Trans. Veh. Technol..

[46] Goodman N.A., Venkata P.R., Neifeld M.A. (2007). Adaptive Waveform Design and Sequential Hypothesis Testing for Target Recognition with Active Sensors. IEEE J. Sel. Top. Signal Process..

